# Planned Distal Pancreatectomy and Splenectomy for Pancreatic Tail Necrosis and Splenic Infarction after Embolization for Gastric Ulcer–Induced Splenic Artery Perforation: A Case Report

**DOI:** 10.70352/scrj.cr.26-0385

**Published:** 2026-10-17

**Authors:** Yasutaka Saito, Kazuki Masiko, Taichiro Ueda, Yoshiaki Hara

**Affiliations:** 1 Emergency Center/Shock and Trauma Center, Nippon Medical School Chiba Hokusoh Hospital, Inzai, Chiba, Japan; 2 Department of Surgery, Masuda Red Cross Hospital, Masuda, Shimane, Japan; 3 Department of Emergency and Critical Care Medicine, Nippon Medical School, Tokyo, Japan

**Keywords:** splenic artery embolization, N-butyl-2-cyanoacrylate, pancreatic tail necrosis, splenic infarction, distal pancreatectomy, planned surgery, gastric ulcer

## Abstract

**INTRODUCTION:**

Splenic artery perforation secondary to gastric ulcer is a rare but life-threatening cause of upper gastrointestinal bleeding. Splenic artery embolization (SAE) can provide rapid hemorrhage control, but delayed ischemic complications involving the pancreatic tail and spleen are uncommon.

**CASE PRESENTATION:**

A woman in her 70s presented with hematemesis and profound hemorrhagic shock. Contrast-enhanced CT showed active extravasation from the splenic artery adjacent to the posterior gastric wall. During emergency SAE, coils were deployed across the bleeding point, but persistent extravasation required additional embolization with a 1:2 mixture of N-butyl-2-cyanoacrylate (NBCA) and lipiodol. Final angiography confirmed hemostasis with preservation of the dorsal and great pancreatic arteries. CT on hospital day 2 showed no apparent pancreatic ischemic change. On day 11, newly developed hypoattenuating areas involving the pancreatic tail and part of the spleen were detected, suggestive of ischemic changes. The patient had no abdominal pain or overt clinical deterioration. Laboratory abnormalities that had improved by day 8 showed a secondary increase on day 11, with a white blood cell count of 12200/μL, C-reactive protein level of 3.90 mg/dL, and serum amylase level of 456 U/L. Planned distal pancreatectomy and splenectomy were selected after individualized assessment of the pancreatic and splenic ischemic changes, together with a residual splenic hilar aneurysmal lesion and suspected posterior gastric wall penetration. Pathological examination confirmed pancreatic necrosis and splenic infarction. No postoperative pancreatic fistula or other major postoperative complication occurred, and the patient was discharged on hospital day 38 after rehabilitation.

**CONCLUSIONS:**

NBCA-assisted SAE can achieve rapid hemostasis in life-threatening splenic arterial bleeding, but post-embolization surveillance remains important. This case illustrates that management of post-SAE ischemic changes should be individualized and that planned surgery in a clinically stable patient should be based on the overall clinical and anatomical context.

## Abbreviations

DP
: distal pancreatectomy
NBCA
: N-butyl-2-cyanoacrylate
SAE
: splenic artery embolization

## INTRODUCTION

Splenic artery perforation or pseudoaneurysm secondary to gastric ulcer is a rare but potentially fatal cause of upper gastrointestinal bleeding. Previous reports have described gastric ulcer–related splenic arterial lesions treated with transcatheter arterial embolization, with surgery required when embolization was insufficient to achieve durable hemostasis.^1,2)^ When endoscopic hemostasis is unsuitable because of arterial bleeding adjacent to the posterior gastric wall, SAE can provide rapid hemorrhage control.

SAE is generally effective for splenic arterial bleeding, but ischemic complications may occur depending on the embolization site, embolic material, and individual vascular anatomy.^3)^ NBCA is a useful liquid embolic agent when coil embolization alone is insufficient; however, its strong embolic effect may increase the risk of ischemic complications in adjacent organs.^4)^ Splenic infarction and abscess are recognized complications after SAE, whereas pancreatic tail necrosis is rare but potentially serious.^5)^

The optimal management of pancreatic tail and splenic ischemic injury after SAE remains unclear, particularly when ischemic changes are detected before infection or clinical deterioration. We report a rare case of pancreatic tail necrosis and splenic infarction following SAE with NBCA for gastric ulcer–induced splenic artery perforation, managed by planned DP and splenectomy.

## CASE PRESENTATION

A woman in her 70s with no significant past medical history presented with hematemesis. She had been intermittently taking loxoprofen. During ambulance transport, her level of consciousness deteriorated, and agonal breathing was observed just before arrival.

On arrival (day 1), she was in profound shock, presenting with a Glasgow Coma Scale score of E1V1M1, marked pallor, and non-palpable radial pulses. After intubation and transfusion of 6 units of red blood cells and plasma, contrast-enhanced CT revealed posterior gastric wall thinning and extravasation from the adjacent splenic artery, consistent with splenic artery perforation (**Fig. 1A**), along with a 25-mm saccular aneurysm at the splenic hilum. Laboratory tests showed severe anemia (hemoglobin 5.6 g/dL), leukocytosis (white blood cell count 15200/μL), coagulopathy (prothrombin time–international normalized ratio 1.5), and metabolic acidosis (pH 7.231, lactate 13.7 mmol/L). During the urgent SAE, a microcatheter was advanced beyond the arterial disruption. Coils were deployed from the distal to the proximal side across the bleeding point; however, extravasation persisted (**Fig. 1B**). A 1:2 mixture of NBCA and lipiodol was therefore injected through the microcatheter positioned within the coil mass. Final angiography confirmed cessation of extravasation and showed preservation of the dorsal pancreatic artery and great pancreatic artery (**Fig. 1C**).

**Fig. 1:**
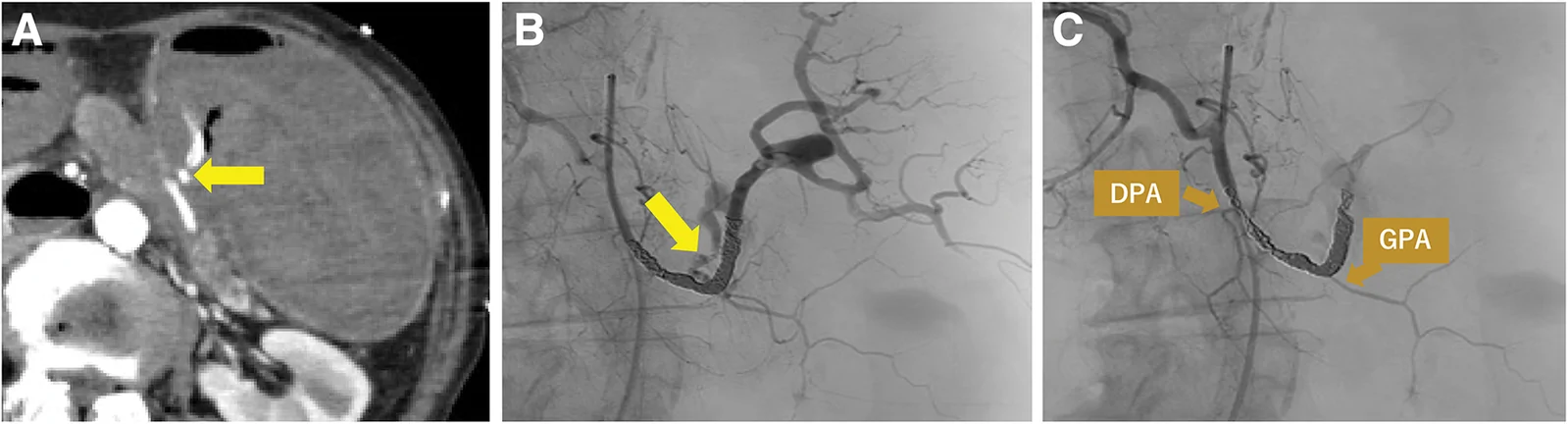
Initial CT and angiographic findings. (**A**) Contrast-enhanced CT on hospital day 1 shows active extravasation from the splenic artery adjacent to the posterior wall of the stomach (arrow). (**B**) Angiography after distal-to-proximal coil embolization across the bleeding point shows persistent extravasation (arrow). (**C**) Final angiography after additional embolization with a 1:2 mixture of NBCA and lipiodol shows complete hemostasis, with preservation of the DPA and GPA. DPA, dorsal pancreatic artery; GPA, great pancreatic artery; NBCA, N-butyl-2-cyanoacrylate

The patient stabilized and was extubated on day 2. CT performed on day 2 showed no apparent ischemic change in the pancreatic tail (**Fig. 2A**). Follow-up CT on day 11 showed newly developed hypoattenuating areas involving the pancreatic tail and part of the spleen, suggestive of ischemic changes (**Fig. 2B** and **2C**). No pancreatic enlargement, peripancreatic fat stranding, peripancreatic fluid collection, or gas formation was observed. She had no abdominal pain or overt clinical deterioration. Serial body temperature and laboratory data from day 1 to day 13 are summarized in **Table 1**. After an initial inflammatory and biochemical response, the white blood cell count, C-reactive protein level, and serum amylase level had decreased to 6800/μL, 3.09 mg/dL, and 93 U/L, respectively, by day 8. On day 11, these values increased to 12200/μL, 3.90 mg/dL, and 456 U/L, respectively, while the maximum temperature was 37.7°C. On day 13, the white blood cell count was 8600/μL, C-reactive protein level 5.20 mg/dL, serum amylase level 247 U/L, and preoperative maximum temperature 36.8°C. Serum lipase was not measured. Thus, mild leukocytosis and C-reactive protein elevation were present on day 11, but there were no overt clinical signs of infection, ongoing bleeding, or acute abdominal deterioration. The pancreatic-tail and splenic ischemic changes coexisted with a residual 25-mm splenic hilar aneurysmal lesion and suspected penetration of the posterior gastric wall. These findings were considered collectively, and planned surgery was selected as an individualized treatment strategy.

**Fig. 2:**
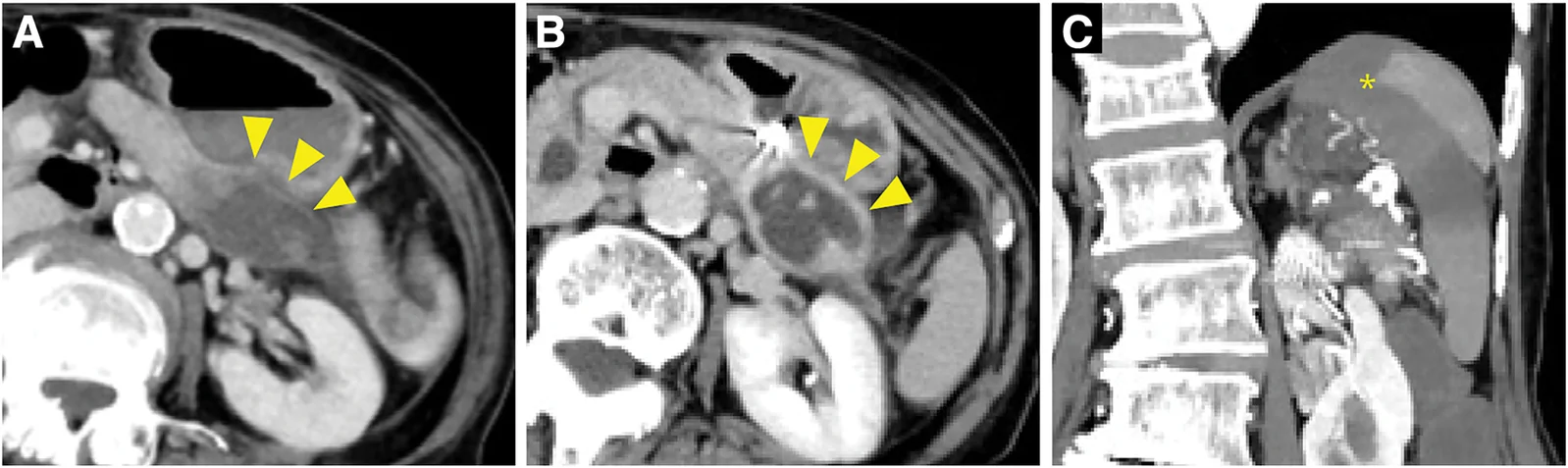
Evolution of pancreatic and splenic ischemic changes. (**A**) Contrast-enhanced CT on hospital day 2 shows no apparent ischemic change in the pancreatic tail. (**B**) Contrast-enhanced CT on hospital day 11 shows a newly developed hypoattenuating area in the pancreatic tail (arrowheads), suggestive of ischemic change. (**C**) Coronal contrast-enhanced CT on hospital day 11 shows a hypoattenuating area in part of the spleen (asterisk), suggestive of splenic ischemia. Pancreatic necrosis and splenic infarction were subsequently confirmed by pathological examination.

**Table 1:** Serial clinical and laboratory course from hospital day 1 to day 13

Hospital day	Maximum temperature (°C)	WBC (/μL)	CRP (mg/dL)	Serum amylase (U/L)	Key clinical event
1	37.1	15200	2.23	78	Hemorrhagic shock; SAE
3	38.1	19900	9.99	308	
4	37.8	14100	10.48	107	
8	37.0	6800	3.09	93	Clinical improvement
11	37.7	12200	3.90	456	CT: newly developed pancreatic-tail and splenic ischemic changes
13	36.8*	8600	5.20	247	Planned surgery

*Preoperative maximum temperature on hospital day 13.

CRP, C-reactive protein; SAE, splenic artery embolization; WBC, white blood cell count

On day 13, open DP and splenectomy were performed. The necrotic pancreatic tail was clearly demarcated. Because ulcer penetration had produced a posterior gastric wall defect with dense adherence to the pancreatic tail, partial resection of the posterior gastric wall was required during en bloc resection (**Fig. 3A** and **3B**). Histopathological examination demonstrated pancreatic parenchymal necrosis and splenic infarction. Embolic material was identified within vessels, and no malignancy was present. The original pathological report did not provide a detailed semiquantitative assessment of viable pancreatic tissue, inflammatory cell infiltration, or vascular thrombosis. No postoperative pancreatic fistula or other major postoperative complication occurred. Drain-fluid amylase was measured serially and remained low; the highest value was 170 U/L on POD 1. The left subphrenic drain was removed on POD 7 and the drain adjacent to the pancreatic stump on POD 11. The patient required continued rehabilitation for deconditioning and recovery of ADL and was discharged on day 38 (POD 25).

**Fig. 3:**
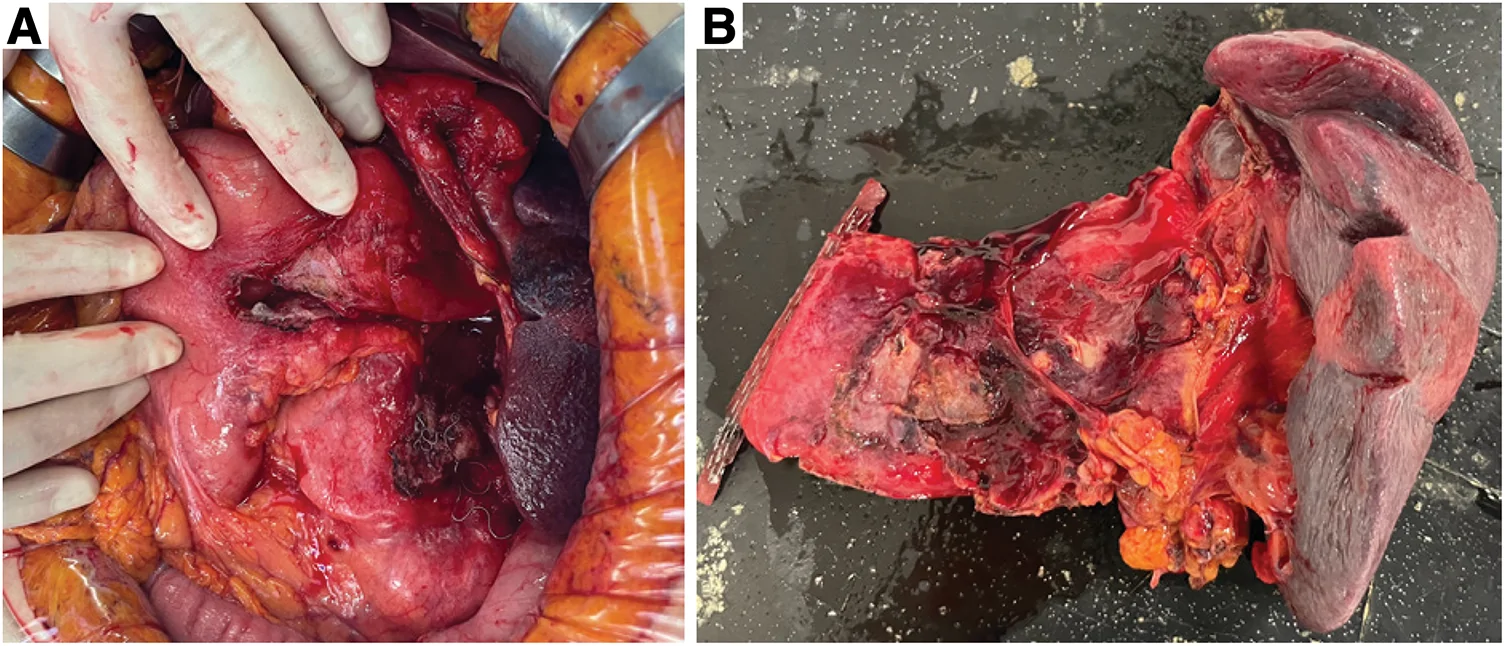
Intraoperative and gross findings. (**A**) Intraoperative findings show a clearly demarcated necrotic area in the pancreatic tail. (**B**) Gross examination of the resected specimen shows the spleen on the right and the distal pancreas on the left, with necrotic changes involving the pancreatic tail.

## DISCUSSION

In this case, newly developed ischemic changes involving the pancreatic tail and part of the spleen were detected on follow-up imaging after successful NBCA-assisted SAE for gastric ulcer–induced splenic artery perforation. The patient had no abdominal pain, ongoing bleeding, or overt clinical signs of infection. The laboratory abnormalities that had improved by day 8 showed a secondary increase on day 11, coinciding with the newly detected CT abnormalities, although they did not indicate clinical deterioration. Planned DP and splenectomy were performed after individualized assessment of the combined local findings. Pathological examination subsequently confirmed pancreatic necrosis and splenic infarction. This case highlights that successful endovascular hemostasis requires subsequent surveillance and that management of post-embolization ischemic injury should be individualized.

Gastric ulcer–related splenic artery pseudoaneurysm or perforation is a rare but potentially fatal cause of upper gastrointestinal bleeding. Previous reports have described lesions treated by transcatheter arterial embolization, with surgery required when embolization failed to provide durable hemostasis.^1,2)^ In the present case, the bleeding source was adjacent to the posterior gastric wall, making endoscopic hemostasis unsuitable. A microcatheter was advanced beyond the disruption, and coils were deployed from distal to proximal across the bleeding point, constituting an isolation strategy. Persistent extravasation after coil placement prompted additional NBCA embolization within the coil mass. Thus, NBCA was used as a rescue adjunct rather than as a general substitute for isolation embolization.

The pancreatic tail is usually supplied through multiple branches and intrapancreatic arterial arcades arising from the splenic artery, including the dorsal, great, and transverse pancreatic arteries. Therefore, pancreatic tail necrosis after SAE is uncommon. In this patient, final angiography showed preservation of the dorsal and great pancreatic arteries. Nevertheless, angiographic visualization of these vessels did not necessarily ensure adequate perfusion of the pancreatic tail parenchyma. Possible contributors included compromise of smaller intrapancreatic branches and profound systemic hypoperfusion during hemorrhagic shock; however, the exact mechanism, the distal distribution of NBCA, and the functional adequacy of the collateral circulation could not be determined retrospectively.

Previously reported cases of pancreatic necrosis after SAE are summarized in **Table 2**.^6–10)^ Most occurred after SAE for traumatic splenic injury. One reported patient was managed non-operatively, whereas symptomatic patients with severe abdominal pain, peritonitis, or sepsis frequently underwent DP, often with splenectomy. Thus, pancreatic necrosis after SAE does not invariably mandate surgery. The distribution and severity of pancreatic and splenic ischemic injury, clinical deterioration, evidence of infection, and unresolved local vascular or gastrointestinal lesions may all contribute to individualized management. In contrast to the surgically managed reports, the present case was identified before abdominal symptoms, overt infection, or sepsis developed.

**Table 2:** Reported cases of pancreatic necrosis after SAE

Author, year	Indication/embolization	Timing and presentation	Extent/infection	Management	Outcome
Hamers et al., 2009^6)^	Splenic trauma; inadvertently extensive proximal SAE	Timing not specified; acute necrotizing pancreatitis	Extent and infection status not specified	Non-operative management	Not reported
Khurana et al., 2011^7)^	Splenic trauma; embolization details not reported	2 days; severe abdominal pain and abdominal distension	Pancreatic tail necrosis; infection status not reported	Laparotomy; DP and splenectomy	Not reported
Paul and Opalek, 2011^8)^	Splenic trauma; proximal SAE; 2 cases	Timing and detailed presentation not reported in the indexed record	Distal pancreatic necrosis; infection status not reported	DP in both cases; splenectomy not reported	Not reported
Talving et al., 2016^9)^	Grade IV splenic trauma; branch embolization with gelatin sponge followed by proximal coils	Next day; severe abdominal pain, tachycardia, distress, and peritoneal signs	Non-perfused pancreatic tail; no preoperative sepsis reported	Laparotomy; DP and splenectomy	Discharged on POD 22 after intra-abdominal abscess, pleural effusion, and pancreatic fistula
Yoo et al., 2023^10)^	Grade IV splenic trauma; proximal SAE	1 week; severe sepsis	Non-perfusion and necrosis of approximately 40% of the pancreas	Laparotomy; DP and splenectomy	Prolonged hospital course with multiple complications; final disposition not reported
Present case	Gastric ulcer–induced splenic artery perforation; distal-to-proximal coils plus NBCA–lipiodol (1:2)	11 days; no abdominal pain, maximum temperature 37.7°C, and mild laboratory abnormalities	Near-complete pancreatic tail ischemia and substantial splenic infarction; no overt clinical infection	Planned DP and splenectomy	Uneventful; discharged on hospital day 38

DP, distal pancreatectomy; NBCA, N-butyl-2-cyanoacrylate; SAE, splenic artery embolization

In conventional necrotizing pancreatitis, delayed intervention and a step-up approach are generally preferred, particularly when infected necrosis is suspected.^11)^ The elevated serum amylase level indicated pancreatic injury. However, the absence of abdominal pain, pancreatic enlargement, peripancreatic fat stranding, fluid collection, and gas formation, together with the newly developed localized hypoattenuating changes in the pancreatic tail after SAE, was considered more consistent with predominantly ischemic pancreatic injury than with the typical clinical and radiological presentation of inflammatory necrotizing pancreatitis. This distinction is not absolute, and ischemic injury may itself trigger secondary inflammation. Histopathology subsequently confirmed pancreatic parenchymal necrosis and intravascular embolic material, although the original report did not provide detailed semiquantitative assessment of viable pancreatic tissue, inflammatory-cell infiltration, or vascular thrombosis.

There are no established objective criteria for prophylactic resection of asymptomatic SAE-associated pancreatic ischemic injury. In this patient, proven infection was not the indication for surgery. The decision was individualized because the newly developed pancreatic-tail and partial splenic ischemic changes coexisted with a residual 25-mm splenic hilar aneurysmal lesion and suspected posterior gastric wall penetration. The exact classification of the hilar lesion as a true aneurysm or pseudoaneurysm could not be determined retrospectively. Neither the aneurysmal lesion nor suspected gastric penetration was regarded as an independent indication for urgent surgery; rather, these findings were considered collectively in selecting planned resection. Endovascular treatment could potentially have addressed the vascular lesion alone but would not have treated the concomitant pancreatic, splenic, and gastric abnormalities. Intraoperatively, the posterior gastric wall defect and dense adherence to the pancreatic tail were confirmed and necessitated en bloc partial gastrectomy. These operative findings supported treatment of the adjacent lesions en bloc but do not establish a general indication for surgery in similar patients.

This report does not establish an indication for early surgery in asymptomatic pancreatic or splenic ischemic lesions after SAE. Conservative management remains a reasonable option in clinically stable patients without infection, ongoing bleeding, or other unresolved local pathology. In the present case, planned surgery was selected on the basis of the combined pancreatic, splenic, vascular, and gastric findings; this individualized decision should not be generalized as a management recommendation. Further accumulation of cases is needed to clarify appropriate indications and timing.

## CONCLUSIONS

Pancreatic tail necrosis and splenic infarction are rare but potentially serious ischemic complications after SAE. NBCA-assisted embolization can achieve rapid hemostasis, but careful post-embolization surveillance remains necessary. Management should be individualized. In this case, planned surgery was selected after consideration of multiple coexisting local findings in a clinically stable patient; these findings should not be regarded as established indications for surgery.

## DECLARATIONS

### Availability of data and materials

The datasets generated and/or analyzed during the current report are not publicly available because they contain patient-related clinical information but are available from the corresponding author on reasonable request.
